# Pulp chamber temperature rise in light-cure bonding of brackets with and without primer, in intact versus restored teeth

**DOI:** 10.1590/2177-6709.28.2.e2321167.oar

**Published:** 2023-06-05

**Authors:** Gabriela Cenci SCHMITZ, Fernanda de Souza HENKIN, Mauricio MEZOMO, Mariana MARQUEZAN, Gabriela BONACINA, Maximiliano Schünke GOMES, Eduardo Martinelli Santayana de LIMA

**Affiliations:** 1 Pontifícia Universidade Católica do Rio Grande do Sul (PUCRS), Escola de Ciências da Saúde e da Vida, Programa de Pós-Graduação em Odontologia (Porto Alegre/RS, Brazil).; 2 Universidade Federal de Santa Maria, Escola de Odontologia, Departamento de Estomatologia (Santa Maria/RS, Brazil).; 3 Centro Médico e Odontológico da Polícia Militar do Rio Grande do Sul (Porto Alegre/RS, Brazil).

**Keywords:** Dental cements, Orthodontic brackets, Dental pulp, Orthodontics

## Abstract

**Objective:**

To evaluate the pulp chamber temperature rise (PCTR) in light-cure bonding of brackets with and without primer, in intact and restored mandibular central incisors (M1), maxillary first premolars (Mx4), and mandibular third molars (M8).

**Material and Methods:**

Ninety human teeth were included: M1 (n=30), Mx4 (n=30), and M8 (n=30). Light-cure bonding of brackets was performed in intact (n=60) and restored (n=30) teeth, with primer (n=60) or without (n=30) primer. PCTR was defined as the difference between initial (T0) and peak temperatures (T1), recorded with a thermocouple during light-cure bonding. Differences on PCTR between bonding techniques (primer *vs.* no primer), teeth types (M1 *vs.* Mx4 *vs.* M8), and teeth condition (intact *vs.* restored) were estimated by ANCOVA, with α=5%.

**Results:** PCTR was significantly higher with the use of primer (2.05 ± 0.08^o^C) than without primer (1.65 ± 0.14^o^C) (*p*=0.02), and in M1 (2.23 ± 0.22^o^C) compared to Mx4 (1.56 ± 0.14^o^C) (*p*<0.01). There was no difference in the PCTR in M8 (1.77 ± 0.28^o^C) compared to M1 or Mx4 (*p*>0.05), and no difference between intact (1.78 ± 0.14^o^C) and restored (1.92 ± 0.08^o^C) teeth (*p*=0.38). There was no influence of dentin enamel thickness in the PCTR (*p*=0.19).

**Conclusion:**

PCTR was higher in light-cure bonding of brackets with primer, especially in M1. Light-cure bonding seems less invasive without primer.

## RESUMO

**Objetivo:**

Comparar o aumento na temperatura da câmara pulpar (ATCP) na colagem de braquetes com fotoativação usando ou não *primer*, em dentes hígidos *versus* dentes restaurados, em incisivos centrais inferiores (ICI), primeiros pré-molares superiores (1PMS) e terceiros molares inferiores (3MI).

**Métodos:**

Foram incluídos noventa dentes humanos: ICI (n=30), 1PMS (n=30) e 3MI (n=30). A colagem de braquetes com fotoativação foi realizada em dentes hígidos (n=60) ou dentes restaurados (n=30), com *primer* (n=60) ou sem (n=30) *primer*. O ATCP foi definido como a diferença entre as temperaturas inicial (T0) e máxima (T1) registradas com um termômetro durante a colagem com fotoativação. As diferenças no ATCP entre as técnicas de colagem (com *primer vs.* sem *primer*), diferentes dentes (ICI*vs.*1PMS*vs.*3MI) e condição dos dentes (hígidos *vs.* restaurados) foram estimadas por ANCOVA, com α=5%.

**Resultados:**

O ATCP foi significativamente maior com o uso de *primer* (2,05 ± 0,08^o^C) do que sem *primer* (1,65 ± 0,14^o^C) (*p*=0,02), e no ICI (2,23 ± 0,22^o^C) do que no 1PMS (1,56 ± 0,14^o^C) (*p*<0,01). Não houve diferença no ATCP do 3MI (1,77 ± 0,28^o^C) em comparação com ICI ou 1PMS *(p*>0,05), e não houve diferença entre dentes hígidos (1,78 ± 0,14^o^C) e restaurados (1,92 ± 0,08^o^C) (*p*=0,38). Não houve influência da espessura da dentina e do esmalte no ATCP (*p*=0,19).

**Conclusão:**

O ATCP foi maior na colagem de braquetes com fotoativação usando *primer*, principalmente no ICI. A colagem com fotoativação parece ser menos invasiva sem *primer*.

## INTRODUCTION

Clinical dental procedures can lead to pulp chamber temperature rise (PCTR).^1^ ^-^ ^4^ Minor temperature elevations cause none or mild harm that can be reversed by means of physiological reactions of pulp tissues. Temperature increases above 5.5°C can represent a high risk of pulp inflammation and consequent pulp necrosis.^5^ ^,^ ^6^ Light-cure bonding of brackets generates a wide range of heat variations, usually related to different light sources, exposure times, adhesive resin thickness, and exothermic reactions.^7^ ^-^ ^10^ Even within the limits for irreversible damage in the pulp tissue, PCTR is undesired. ^2,5^

Standard bonding of brackets follows two light-curing steps: one for the primer, and another for the resin adhesive. The primer can enhance shear bond strength and offer better protection for etched enamel prisms, due to its thinner viscosity.^11^ On the other hand, bracket bonding without the use of primer takes shorter time and decreases the exposure to moisture, which is a risk factor for bond failure. *In vitro* studies found equal shear bond strength in brackets bonded either using primer or not using primer.^12^ ^,^ ^13^ Likewise, clinical studies reported no differences in bond failures between brackets bonded with or without the use of primer.^14^ ^-^ ^16^ One-step bonding without primer saves time for light curing, and avoids cumulative heat that can lead to PCTR.^17^ Moreover, bracket bonding without primer tends to reduce the amount of resin adhesive remaining after debonding.^16^ Shorter time for resin adhesive removal prevents a new episode of PCTR.^4^

Tooth conditions may play a role in the transfer of heat to the pulp chamber. Teeth have poor thermal conductivity; hence, the microstructure of the dentin-enamel junction functions to protect the pulp against temperature changes.^18^ Thicker layers of dentin-enamel tissues appear to prevent PCTR.^19^ Thus, intact teeth might be less vulnerable to PCTR than restored teeth. Thermal conductivity of composite resins can induce a more aggressive reaction of pulp tissues.^18^

Thus, the present study aimed to evaluate the PCTR during light-cure bonding of brackets, with and without the use of primer, in mandibular central incisors (M1), maxillary first premolars (Mx4), and mandibular third molars (M8), under both intact and restored conditions. The null hypothesis tested was that PCTR would not present significant differences in relation to bonding techniques, tooth types, or tooth conditions.

## MATERIAL AND METHODS

This study was approved by the Committee of Ethics and Research of the Pontifical Catholic University of Rio Grande do Sul (PUCRS) (CAAE: 32437314.0.0000.5336). Human teeth were obtained from invited patients who signed an informed consent form, and were extracted due to therapeutic reasons in the Service of Dentistry at the PUCRS School of Life and Health Sciences. All teeth were donated for research purposes.

M1, Mx4, and M8, with intact buccal surfaces and intact pulp chambers were included. After inspection, teeth with dentin lesions, large cavities, or surgical damage were excluded from the study. Ninety human teeth met the inclusion criteria and were stored in saline, at room temperature, up to four months, until the experiment. 

Ten samples of each tooth type were randomly selected, prepared with dental cavities, subsequently restored with composite resin, then brackets were bonded using primer (n = 30). The other 20 samples of each tooth type were divided in two groups: brackets bonded using primer (n = 30) and without primer (n = 30) (Table 1). Sample size calculation resulted in 30 samples per group to detect a difference of 0.15^o^C (2.14 ± 0.18^o^C)^19^ between groups, with a power of 90%, and significance level of 5%.

**Table 1:** Sample distribution according to the experimental groups.

	Tooth type (n = 90)		
	M1 n = 30	Mx4 n = 30	M8 n = 30
Restored + Primer n= 30	10	10	10
Intact + Primer n= 30	10	10	10
Intact + No Primer n = 30	10	10	10

M1 = mandibular central incisor; Mx4 = maxillary first molar; M8 = mandibular third molar.

### CAVITY PREPARATION AND COMPOSITE RESTORATION

M1, Mx4, and M8 were prepared and restored as the Institution’s general guidelines. M1 were prepared with class V cavities, using a high-speed diamond bur 1014 (Jet, Vancouver, BC, Canada), and cavities had a depth of 1.5 mm and width of 2/3 the buccal surface. Mx4 and M8 were prepared with class II mesial-occlusal-distal (MOD) cavities, using a high-speed carbide bur 245 (Jet), and cavities had an occlusal depth equal to the height of the bur head, proximal depth 2 mm below the marginal ridges, mesial width and distal width of 2/3 the proximal surface, and occlusal width of 50% the distance between buccal and lingual cusps. 

The cavities were etched with 37% phosphoric acid for 20 seconds, rinsed with water for 20 seconds, and dried with water-free air jet. Dental restorations were performed with Adapter™ Single Bond Plus - Z250 XT A3 composite (3M ESPE, Saint Paul, MN, USA), in two steps: At first, the primer was light-cured; then, Z250 XT A3 composite (3M ESPE) was added, adapted, and light-cured, following the manufacturer’s instructions.

### ADAPTATION OF THE THERMOCOUPLE FOR THERMAL ANALYSIS

**Figure 1:**
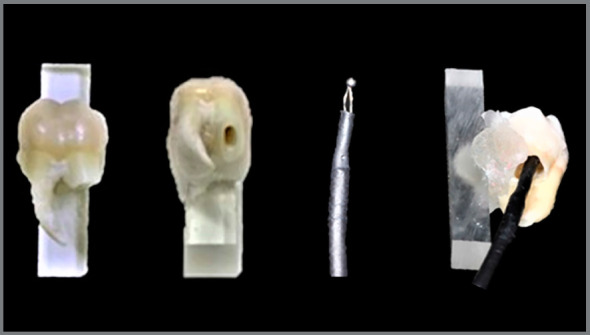
Specimen and thermocouple device inserted in the tooth root.

PCTR was recorded with a 1.6-mm K-type thermocouple (MTK-01, Minipa, São Paulo, SP, Brazil), temperature amplitude of 40-204°C, and accuracy of ±2.2°C. The device was inserted in the pulp chamber via root access (Fig 1). 

The distal root of M8 and buccal root of Mx4 were sectioned at 5 mm from the cementum-enamel junction. The pulp chambers were cleaned using a dentin excavator, irrigated for 60 seconds with 2% sodium hypochlorite solution, rinsed with distilled water, and dried by oil-free air jet. The specimens were fixed in a prefabricated device, using self-curing acrylic resin, with the buccal surfaces exposed, to bracket bonding, and the root access unobstructed (Fig 1). 

**Figure 2:**
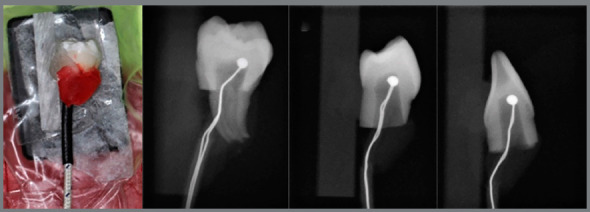
Thermocouple positioned in M8, Mx4, and M1.

The thermocouple was placed against the buccal surface of the pulp chamber, stabilized with utility wax, and connected to a previously calibrated digital thermometer (HI 935005, Hanna, São Paulo, SP, Brazil). The thermocouple position was standardized at the central part of the pulp chamber’s buccal wall, with the aid of digital X-rays (Gnatus Timex 70 E, Ribeirão Preto, SP, Brazil) obtained at a distance of 30 cm (exposure time of 0.1 seconds). The specimens were fixed in the X-ray sensor (Cygnus Ray, Tampa, FL, USA) with double-sided tape, with the proximal surfaces parallel to the sensor and perpendicular to the X-ray beam. Radiographic analysis (Cygnus Media 3.0) assured proper placement of the thermocouple (Fig 2). The specimens were then fixed in a glass plate using double-sided tape.

### ORTHODONTIC BONDING

Standard stainless steel brackets (0.022-in, American Orthodontics, Sheboygan, WI, USA) were bonded to the buccal surface of the teeth with Transbond XT (3M Unitek, Monrovia, CA, USA). Enamel etching was performed with 37% phosphoric acid for 30 seconds, teeth were rinsed with water for 30 seconds, and dried with water-free air jet for 20 seconds, at a distance of 15 cm. In the samples of the group with primer, a thin layer of primer was applied and light-cured with LED (Abzil 3M, Ribeirão Preto, SP, Brazil), with 420-480 nm wavelength, 1200 ± 20 nm/cm² light intensity, in continuous mode, for 10 seconds, at a distance of 5 mm. 

The brackets with resin adhesive were pressed against the center of the buccal surfaces of the teeth with 454 gf of force, which was measured with a Gilmore needle. The resin adhesive was light-cured with the same LED (Abzil 3M), for 20 seconds (10 seconds in the mesial and 10 seconds in the distal of the bracket), at a distance of 5 mm.

### PULP CHAMBER TEMPERATURE

**Figure 3:**
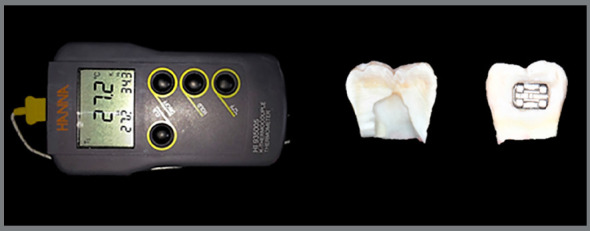
Temperature records and a split specimen.

Temperature (°C) in the pulp chamber was recorded from the onset until 20 seconds after the light-cure bonding of brackets. The thermometer recorded the initial (T0) and peak temperatures (T1). PCTR was defined as the difference between records (T1 − T0) (Fig 3). Specimen preparation and temperature assessments were performed in a random sequence, at room temperature.

### ENAMEL-DENTIN THICKNESS

The teeth were cut in a mesial-distal direction using a diamond disk (1802.7016, KG Sorensen, Cotia, SP, Brazil) under refrigeration (Fig 3). The enamel-dentin thickness of the pulp chamber buccal wall was measured using a digital caliper (Starret, Athol, MA, USA), with 0.01-mm accuracy. This measurement was performed in order to control the possible effect of this covariate on the measured temperature.

### STATISTICAL ANALYSIS

The Kolmogorov-Smirnov test and Levene test assessed the data distribution. Analysis of covariance (ANCOVA) was performed with a robust standard error, i.e., a covariance analysis of four factors (bonding technique, tooth type, tooth condition, and enamel-dentin thickness), with one factor being continuous (thickness). Data were analyzed in the SPSS software (version 18.0, Chicago, IL), at 5% significance level. 

## RESULTS

**Figure 4:**
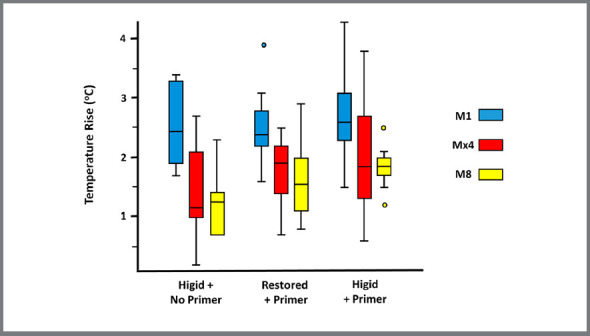
Box-plot of PCTR distribution.

PCTR occurred in all specimens (overall mean = 1.94°C; range = 0.2-4.3°C) (Fig 4). PCTR showed statistically significant differences between bonding techniques (with primer *vs.* without primer; *p* = 0.02) and between tooth types (M1 *vs.* Mx4 *vs.* M8; *p* < 0.05) (Table 2).

**Table 2:** Descriptive statistics of pulp chamber temperature rise (PCTR). Interactions by bonding technique, teeth type, tooth condition, and thickness.

Factor		PCTR			p
		n	Mean ± SD (^o^C)	95% confidence interval	
Bonding technique	With primer	60	2.05 ± 0.08	1.89 - 2.22	0.02*
	No primer	30	1.65 ± 0.14	1.37 - 1.93	
Tooth type	M1	30	2.23 ± 0.22^A^	1.8 - 2.65	< 0.01*
	Mx4	30	1.56 ± 0.14^B^	1.3 - 1.83	
	M8	30	1.77 ± 0.28^AB^	1.22 - 2.32	
Tooth condition	Intact	60	1.78 ± 0.14	1.5 - 2.05	0.38
	Restored	30	1.92 ± 0.08	1.76 - 2.09	
Tooth thickness	Buccal	90	2.88 ± 0.54	2.12 - 3.88	0.19

Analysis of covariance (*p* < 0.05). ^o^C = Celsius degrees; SD = standard deviation; * Statistical significance; different letters by line = significant difference.

PCTR was statistically higher with primer (2.05 ± 0.08°C) than without primer (1.65 ± 0.14°C) (*p* = 0.02). M1 showed PCTR (2.23 ± 0.22°C) statistically higher than Mx4 (1.56 ± 0.14°C) (*p*< 0.01), whereas PCTR in M8 (1.77 ± 0.28°C) showed no differences when compared with that in M1 or Mx4 (*p* > 0.05) (Table 2).

PCTR in intact (1.78 ± 0.14°C) and restored teeth (1.92 ± 0.08°C) showed no statistically significant difference (*p* = 0.38). PCTR showed no significant relationship with dentin-enamel thickness (*p* = 0.19) (Table 2).

## DISCUSSION

This study evaluated PCTR during light-cure bonding of brackets in human teeth, comparing bonding techniques (with primer *vs.* without primer), tooth types (M1 *vs.* Mx4 *vs.* M8), and tooth conditions (intact *vs.* restored). The null hypothesis was partially rejected, since PCTR was statistically different according to the technique (*p* = 0.02) and tooth type (*p* < 0.01) and not significantly different according to tooth condition (*p* = 0.38).

PCTR can be measured by different methods such as using an infrared camera,^8^ calorimeter, and thermocouple.^4^ ^,^ ^20^ ^-^ ^22^ Thermocouples are reliable sensors, as they are composed of a transducer with two dissimilar pieces of wire joined at one end.^20^ The device is manufactured using noble metal and platinum (types R, S, B) or using base metal and nickel (types K, J, N, E, T). The use of a K-type thermocouple is justified by its size, allowing the insertion into the root canal, as previously described.^4^ ^,^ ^9^ ^,^ ^22^ PCTR measurement has a greater accuracy using thermocouples than using other methods,^23^ due to the possibility of ensuring the proper position in the pulp chamber, using radiographs.^6^ ^,^ ^24^

Light-cure bonding of brackets was carried out with the same LED source, in continuous mode, and maximum intensity. LED in continuous mode produces less heat than in ramp or pulsatile modes.^24^ PCTR continued for 20 seconds after the end of the light-curing process, due to possible cumulative heat in the pulp chamber.^17^

Heat is the most severe stress supported by the dentin during dental procedures, inducing a concomitant response in the pulp tissues.^6^ ^,^ ^25^ The main effects of heat on biological tissues are vasodilatation, exudation, and coagulative necrosis. Strong and rapid alternate expansion and contraction of intra-tubular fluid can damage the odontoblasts.^6^

The present results showed that light-cure bonding of brackets without primer is less invasive than with primer (1.65 ± 0.14°C *vs.* 2.05 ± 0.08°C). A possible reason for such difference is the cumulative heat generated by the two-step light-curing, which takes 50% longer time of light exposure (30 seconds) than the one-step light-curing (20 seconds).^18^ ^-^ ^19^

A greater PCTR in M1 (2.23 ± 0.22°C) is consistent with previously described results.^26^ In addition, the different PCTR between tooth types may be related to the smaller volumetric area of the pulp chamber in M1 than in Mx4. One could say that heat dissipates faster in a larger room. On the contrary, an explanation for PCTR of M8 (1.77 ± 0.28°C) halfway between M1 and Mx4 is a thinner layer of dentin tissue, associated with a large pulp chamber.^25^

Dentin-enamel thickness was smaller in M1 (2.1 mm) and greater in M8 (3.9 mm), with no statistically significant difference between groups (*p* > 0.05) (Table 2), probably due to the morphologic variation of M8. Previous studies have found that greater enamel-dentin thickness prevents PCTR, due to the dentin’s thermal insulation role.^1^ ^,^ ^8^ ^,^ ^18^ ^,^ ^20^ ^,^ ^27^ ^,^ ^28^ However, in this study, dentin-enamel thickness had a low influence on PCTR. The divergence of outcomes may be explained by the analysis, which was performed without separation according to different tooth types.

In the present study, restored teeth were bonded with primer and two steps of light-curing, which is a more invasive procedure. Despite that, PCTR was not significantly different (*p*=0.38) between intact teeth and restored teeth (Table 2). Thus, including an additional group with restored teeth and no primer proved dispensable.

The thickness of adhesive layer between enamel and brackets was a confounding factor, which was controlled by a Gilmore needle. All dental restorations were performed with the same composite resin, in order to eliminate differences due to dental materials. Light-colored composite resins may show a greater temperature rise than darker ones during light-curing. Lighter shades favor light transmission, whereas darker shades are prone to light absorption.^28^

This study raised a clinical implication: light-cure bonding of brackets with primer caused a PCTR higher than 3°C in 25% of M1 (maximum = 4.3°C). When the primer was not used, PCTR was lower than 3.4°C in 100% of M1 (Fig 4). In this sample, one-step light-cure bonding of brackets was less invasive, especially in M1. This is a relevant information due to other PCTR events during orthodontic treatment, such as brackets re-bonding, resin adhesive removal, and enamel polishing.^4^ ^,^ ^28^

A limitation of this *in vitro* study is that heat conduction due to blood circulation inside the tooth and fluid movement inside the dentinal tubules was not considered.^20^ In addition, the underlying periodontal tissues promotes heat dissipation *in vivo*, thus controlling the increase in pulp chamber’s temperature.^27^ Histopathological studies would enhance the current knowledge of thermal injury to the pulp during orthodontic bonding, to avoid unwanted outcomes such as pulpitis or pulp necrosis.

## CONCLUSIONS

PCTR during light-cure bonding of brackets was higher with primer than without primer, was higher in M1 than in Mx4, showed no difference in M8 compared to M1 or Mx4, showed no difference between intact and restored teeth, and was not related to dentin-enamel thickness.
